# Dr. Slich's Discovery

**Published:** 1891-07

**Authors:** 


DR. SLICH'S DISCOVERY.—Dr. C. L. Slich, of Berlin, has made an interesting discovery. He was conducting experiments with a view to determining how weak a solution of cocaine would prove efficacious as a local anæsthetic in surgical operations, when he accidentally discovered that simple water injected under the skin with a syringe renders the flesh at that point insensible to pain The effect of water is to create a slight swelling, resembling that caused by the sting of a gnat. The space marked by the swelling remains insensible for some time. The method of procedure is very simple. The skin at the point where the injection is to be made is first made asceptic; then the point of a pravaz syringe filled with distilled water is inserted. The syringe is slowly emptied and a white blister appears. The size of the swelling is according to the amount of water used.

Dr. Slich made use of this discovery in case of a huge carbuncle in the thigh. After the injection of water the doctor laid the carbuncle open by cross incisions, eight centimetres in length, and took out the dead tissue, the patient declaring that the operation gave slight pain. The treatment had no ill-effect in healing of the wound. In the case here mentioned the cuts immediatedly healed and perfectly.

---

